# Environmental Drivers of Free-Living vs. Particle-Attached Bacterial Community Composition in the Mauritania Upwelling System

**DOI:** 10.3389/fmicb.2018.02836

**Published:** 2018-11-23

**Authors:** Jennifer Bachmann, Tabea Heimbach, Christiane Hassenrück, Germán A. Kopprio, Morten Hvitfeldt Iversen, Hans Peter Grossart, Astrid Gärdes

**Affiliations:** ^1^Leibniz Centre for Tropical Marine Research (ZMT), Bremen, Germany; ^2^Faculty of Biology and Chemistry (FB2), University of Bremen, Bremen, Germany; ^3^Max Plank Institute for Marine Microbiology, Bremen, Germany; ^4^Helmholtz Young Investigator Group SEAPUMP, Alfred Wegener Institute for Polar and Marine Research, Bremerhaven, Germany; ^5^Center for Marine Environmental Sciences (MARUM), University of Bremen, Bremen, Germany; ^6^Leibniz-Institute of Freshwater Ecology and Inland Fisheries (IGB), Berlin, Germany; ^7^Institute of Biochemistry and Biology, University of Potsdam, Potsdam, Germany

**Keywords:** prokaryotes, biodiversity, microbial ecology, alpha diversity, Bray Curtis dissimilarity, temperature, salinity, 16S rRNA Illumina amplicon sequencing

## Abstract

Saharan dust input and seasonal upwelling along North–West Africa provide a model system for studying microbial processes related to the export and recycling of nutrients. This study offers the first molecular characterization of prokaryotic particle-attached (PA; >3.0 μm) and free-living (FL; 0.2–3.0 μm) players in this important ecosystem during August 2016. Environmental drivers for alpha-diversity, bacterial community composition, and differences between FL and PA fractions were identified. The ultra-oligotrophic waters off Senegal were dominated by Cyanobacteria while higher relative abundances of Alphaproteobacteria, Bacteroidetes, Verrucomicrobia, and Planctomycetes (known particle-degraders) occurred in the upwelling area. Temperature, proxy for different water masses, was the best predictor for changes in FL communities. PA community variation was best explained by temperature and ammonium. Bray Curtis dissimilarities between FL and PA were generally very high and correlated with temperature and salinity in surface waters. Greatest similarities between FL and PA occurred at the deep chlorophyll maximum, where bacterial substrate availability was likely highest. This indicates that environmental drivers do not only influence changes among FL and PA communities but also differences between them. This could provide an explanation for contradicting results obtained by different studies regarding the dissimilarity/similarity between FL and PA communities and their biogeochemical functions.

## Introduction

Bacteria and archaea, the unseen majority (Whitman et al., 1998), carry out important steps in the biogeochemical cycling of carbon and nutrients, and thus are pivotal for the functioning of marine ecosystems. Many heterotrophic bacteria hydrolyze particulate into dissolved organic matter (OM) (Azam and Malfatti, 2007), which is further hydrolyzed into small molecules for direct uptake by heterotrophic microorganisms. Furthermore, the microbial loop (Azam et al., 1983) converts waste products and other OM into bioavailable organic and inorganic nutrients and subsequently releases them into the surrounding water supporting new biomass production. Thereby, heterotrophic bacteria provide essential nutrients for phytoplankton primary production, which forms the base of any aquatic food web.

Generally, pelagic bacteria can be categorized as truly free-living (FL), truly particle-attached (PA), and bacteria alternating between the two lifestyles (Grossart, 2010). The truly FL bacteria spend their whole life-cycle suspended in the water column, whereas truly PA bacteria remain predominantly associated with various sorts of particles. In contrast, alternating bacteria can constantly attach and detach from particles and hence move between both life-styles. These differences in life-styles are linked to metabolic differences, e.g., PA bacteria generally have a higher capacity to degrade polymeric OM than FL bacteria (Lyons and Dobbs, 2012). Therefore, it is important to distinguish between bacterial life-styles for a better understanding of bacterial community composition (BCC), dynamics, and functions in the ocean.

Particle-attached bacteria are involved in the degradation of sinking particulate OM and thereby affect the efficiency of the biological carbon pump: Previous studies have shown that microbial communities attached to sinking particles typically possess carbon-specific respiration rates of around 0.1–0.2 d^-1^ (Ploug et al., 1999). Hence, their respiration and activity can importantly reduce the amount of organic carbon that is exported to below the thermocline. In this context, it has since been recognized that the capacity for particulate OM degradation depends on the BCC (Enke et al., 2018). Additionally, it has been shown that sinking particles and their attached microbial communities contribute to the vertical connectivity of the BCC in the ocean (Mestre et al., 2018). Thus to understand the functional role of bacteria, knowledge on PA community composition is of profound importance in marine regions characterized by a high carbon export.

Eastern boundary currents, such as the Canary Current, are among the most productive marine regions of the world (Carr, 2002). By taking up large amounts of carbon, the Canary Current system plays an important role in the global export of organic carbon (Arístegui et al., 2009; Fischer et al., 2016). In the southern part of the Canary Current ecosystem, upwelling of nutrients along the coasts of Mauritania and Senegal supports global fisheries by providing suitable spawning grounds for many economically important fish species (Arístegui et al., 2009). Upwelling and surface currents in this region are influenced by changes in trade winds and the migration of the intertropical convergence zone toward the relatively warmer hemisphere between 2°N during boreal winter and 9°N during summer (Schneider et al., 2014). Additionally, Saharan dust input leads to the natural fertilization of this system (Fischer et al., 2009).

Despite the region’s importance for fisheries and the global carbon cycle, the prokaryotic microbial community off the coast of Mauritania and Senegal has received little attention. Dinitrogen fixation by benthic cyanobacteria (Gier et al., 2017) and microbial methanol uptake (Dixon et al., 2013) have been assessed off Mauritania but only one CARD-FISH-based investigation of the whole BCC has been carried out further offshore (Thiele et al., 2015). They observed highest similarities between PA bacteria at depth and FL bacteria from the deep chlorophyll maximum (DCM). High particle export, frequent changes in the intensity of the year-round upwelling and the natural fertilization with Saharan dust (Fischer et al., 2016), render this region a model system for studying microbial processes related to the export and recycling of inorganic nutrients and carbon in the ocean.

Since the micro-scale heterogeneity varies among aquatic habitats, the distribution of specific bacterial species in the ocean is not uniform (Stocker, 2012), whereby the BCC depends on both abiotic (e.g., temperature, pH, etc.) and biotic factors (e.g., algal blooms, zooplankton feces, etc.). It has been demonstrated that in general about 25% of the variation microbial community composition can be explained by environmental parameters (Hanson et al., 2012). Previous studies have correlated bacterial community structures with environmental parameters, such as salinity (Rieck et al., 2015), quantity and quality of inorganic nutrients and OM (Ortega-Retuerta et al., 2013), particle composition (although unsuccessfully; Zhang et al., 2016) as well as chlorophyll *a* (chl*a*) and temperature (Kan et al., 2006). FL and PA bacteria, however, seem to be differentially affected by bulk water parameters, e.g., the larger the particle, the more insensitive the attached community is to changes in abiotic water parameters (Yung et al., 2016). Thereby, the PA community was mainly influenced by substrate availability and particle quality (Yung et al., 2016). Very few studies have assessed the effects of the latter on the similarity/dissimilarity of FL and PA communities, and there has been some debate in the literature about whether FL and PA bacteria are generally similar (Hollibaugh et al., 2000; Ortega-Retuerta et al., 2013) or dissimilar (DeLong et al., 1993; Acinas et al., 1999; Rieck et al., 2015; Zhang et al., 2016).

**FIGURE 1:**
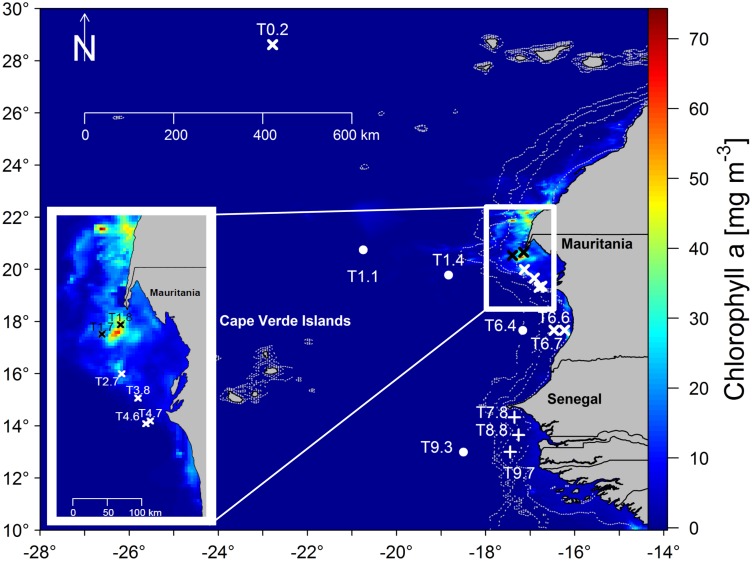
Sampling stations for this project during M129. Sampling was carried out at 16 stations at the surface only (x) surface and DCM (+) and from surface, over DCM to 200 m depth (•). The figure in the lower left corner is an enlargement of the sampling region off Northern Mauritania. Stations T1.7 and T1.8 (black) were situated in the core of the upwelling area, while station T1.4 was within in an anticyclonic eddy. The map was produced from Level 3 Modis Aqua satellite imagery data on monthly chlorophyll a concentrations obtained from https://oceandata.sci.gsfc.nasa.gov/MODIS-Aqua/.

Along the coastlines of Mauritania and Senegal natural horizontal and vertical gradients in environmental variables and substrate availability exist, rendering the North–West African coast an ideal models system for those studies. In the surface waters from Mauritania to Senegal, horizontal gradients of decreasing salinity and inorganic nutrients and increasing temperature result from riverine water discharge and precipitation. Vertically, substrate availability changes from the oligotrophic surface waters off Senegal, over the more productive DCM to 200 m depth, where life almost exclusively relies on sinking OM from the sunlit ocean. The DCM is a prevalent structure of tropical marine environments, occurs at around 20–100 m depth and is characterized by increased concentrations of chl*a*. The DCM often coincides with high primary productivity as environmental conditions are ideal for phytoplankton growth (Eppley et al., 1988; Raimbault et al., 1993; Cullen, 2015). Thus, substrate availability for bacteria may be high at the DCM. However, so far the effect of substrate availability on the BCC in natural environments has received little attention. During the Meteor M129 cruise to the Golf d’Arguin (in Northern Mauritania) and Sine Saloum (Senegal, Figure 1) in July to August 2016, we investigated the BCC off Mauritania and Senegal and the effect of substrate availability on the similarity between FL and PA bacteria by addressing the following research questions:

1. What is the FL and PA microbial community composition off NW Africa, and which conclusions can we draw with regard to the role of PA bacteria in the degradation of organic particles?
2. What are the effects of environmental parameters and substrate availability on the similarity/dissimilarity of FL and PA bacterial communities in this region?

We hypothesize that bacteria, which are adapted to (ultra-)oligotrophic conditions, predominate off Senegal, while the upwelling and potential formation of phytoplankton blooms may stimulate taxa adapted to more mesotrophic conditions off the North–West African coast. Temperature is assumed to be the main environmental driver for changes in overall BCC, and we hypothesize that environmental factors, such as temperature, salinity, and substrate availability, also affect the differences between FL and PA communities, but to a different extent. As we expect the highest substrate availabilities at the depths of the DCM, we hypothesize that FL and PA communities will be most similar there.

## Materials and Methods

The M129 cruise started from the Azores, on July 30 and finished on the Cape Verdes on August 25, 2016. Sampling was carried out along several transects off the coast of Mauritania and Senegal (Figure 1). Sampling stations were distributed over different oceanic regions, including the shelf break and shallow (<50 m) waters closer to the coast. More information about the respective M129 cruise stations (see Supplementary Table 1) is available under https://www.pangaea.de/ddi?retr=events/Meteor_1986/M129.retr&conf=events/CruiseReportHTML.conf&title=Station+list+of+cruise+M129&format=html. Water samples were obtained using a SBE32 rosette water sampler with 24 × 10L Niskin bottles (s/n 0342) from 16 stations over 3 depths: 16 surface samples (ca. 5 m), 7 DCM samples, and 4 samples from 200 m depth (below the thermocline). Water samples were immediately filtered through a 125-μm mesh in order to exclude larger organisms, such as zooplankton. The filtrate was collected in polyethylene canisters and processed under laboratory conditions within 2–3 h.

Physicochemical parameters (temperature, salinity, chl*a*, and turbidity) were measured *in situ* using the Rosette water sampler, which was equipped with a Seabird_electronics SBE9 CTD (s/n 0572) and a digiquartz pressure sensor (s/n 75760) using a double sensor setup.

### Chemical Parameters

Particulate organic carbon (POC) samples were taken at all surface stations. Between 1000 and 5000 mL of marine water were filtered through pre-combusted (450°C for 4 h) and pre-weighted 47 mm GF/F filters (Whatman, Dassel, Germany). Filters were dried at 40°C for at least 24 h and total carbon (TC) and total nitrogen (TN) concentrations were measured on an Elemental Analyzer (EA-3000, EuroVector, Italy). The organic carbon fraction (C_-org_ or POC) was measured after acidification of the filter with 1 N HCl to remove the inorganic carbon. CN ratios were calculated from C_-org_ and TN.

Dissolved inorganic nutrient (NUT) samples were taken in technical triplicates from the POC filtrate and frozen at -20°C until analysis on board. Using a continuous segmented flow auto-analyzer (San System, Skalar, Netherlands) the concentrations of five NUT, i.e., nitrate, nitrite, ammonium, phosphate, and silicate, were determined after Grasshoff et al. (1999).

Technical triplicates of dissolved organic carbon (DOC) concentrations were also obtained from the POC filtrate and preserved frozen at -20°C. Acidified DOC samples were analyzed using the high-temperature combustion method (Dafner and Wangersky, 2002) with a TOC-VCPH autoanalyzer (Shimadzu, Mandel, Canada).

### Plankton Counts

Plankton samples for quantitative analysis were taken at selected surface (T1.1, T0.2, T3.8, T1.4, T4.6, T8.8), DCM (T1.1, T1.4, T7.8, T9.3), and 200 m stations (T1.1, T9.3). Samples were filtered through a 125-μm mesh in order to remove larger organisms and fixed with formalin (2% v/v final concentration). For analysis, 100 mL of the fixed sample were filled into an Utermöhl chamber. Cells were counted after overnight sedimentation at 400× magnification and across 50 random fields. The identified cells were classified into the following plankton groups: diatoms, haptophytes, dinoflagellates, non-flagellated chlorophytes, and other flagellates.

### Bacterial Enumeration

Water samples (50–150 mL) were fixed with formaldehyde (2% v/v) and stored at 4°C for 48 h. PA and FL bacteria were separated via sequential filtration through 3.0 and 0.2 μm (Kegler et al., 2017) Nuclepore TrackEtch polycarbonate membranes (Whatman, Dassel, Germany), respectively. Filters were air-dried and frozen until microscopic analysis (Rieck et al., 2015). 4′,6-Diamidino-2-phenylindole (DAPI; Thermofisher Scientific Inc., Waltham, MA, United States) was diluted in a 3:1 mounting solution (made of Citifluor AF mounting medium; Citifluor Ltd., London, United Kingdom) and Vecta shield (Vector Laboratories Inc., Burlingame, CA, United States) to a concentration of 1 μg mL^-1^. The DAPI/mounting medium solution was directly added onto the filter. FL bacteria were enumerated by using an automatic microbial cell enumeration system. A multipurpose fully automated microscope imaging system (MPISYS) was used for the refined image acquisition. Image selection, cell determination, and enumeration were carried out using the ACMEtool2.0 (Bennke et al., 2016). PA bacteria were manually enumerated with an epifluorescence microscope “Axioskop 40” (Zeiss, Jena, Germany) at 1000× magnifications (Grossart et al., 2005). A minimum of 40 grids (grid size: 15,625 μm^2^) was counted.

### Molecular Analysis of the Microbial Community

FL and PA bacteria were separated as described under “*Bacterial enumeration*” (for filtered volumes see Supplementary Table 2). Filters were frozen on board and transported at -80°C. Storage at the ZMT until further processing was at -20°C. DNA extraction was carried out after Nercessian et al. (2005). Briefly, the phenol–chloroform–isoamylalcohol extraction protocol was adapted for planktonic bacteria as previously proposed by Rieck et al. (2015). Cetyltrimethyl ammonium bromide (CTAB) was used as a complexing agent for polymeric substances. For all samples, the second chloroform–isoamylalcohol washing treatment was skipped. DNA extracts were sent to LGC genomics (Berlin, Germany) for amplicon sequencing of the microbial community. The primer pair Bakt_341F (5′-TCCTACGGGGGCWGCAG-3′) and Bakt_805R (5′-TGACTACHVGGGTATCTAAKCC-3′) was used to target the hypervariable regions V3–V4 of the bacterial 16S rRNA gene (Klindworth et al., 2013). Sequencing was performed on an Illumina MiSeq using V3 Chemistry (Illumina) in a 2× 300 base pair paired-end run.

### Bioinformatic Analyses

Demultiplexing and the removal of primer sequences from the raw paired-end reads with *cutadapt* (Martin, 2011) were performed by LGC genomics. Sequences were further analyzed according to Hassenrück et al. (2016): Sequences were quality trimmed with a sliding widow of four bases and an average quality of 15 using *trimmomatic* v.033 (Bolger et al., 2014). Using *PEAR* v0.9.6 (Zhang et al., 2014), sequences were merged, and *swarm* v2.1.1 was applied to cluster operational taxonomic units (OTUs) (Mahé et al., 2014). Taxonomic classification with SILVA 128 was carried out using the SILVA-ngs pipeline (Quast et al., 2013). Singletons, doubletons, chloroplasts, mitochondria, and OTUs unclassified on phylum level were excluded.

Based on the 16S gene of the chloroplast the presence/absence of eukaryotic phytoplankton genera was inferred (Schmidt et al., 1991; Needham et al., 2017). Chloroplast 16S sequences were aligned against a customized reference database only containing chloroplast sequences from cultivated organisms obtained from the NCBI refseq database (date accessed: June 27, 2017). Sequences with >93% similarity were filtered out for further analysis and the taxonomic path for the best hit was extracted. A detection threshold of at least 10 sequences was chosen to ensure that the sequenced chloroplasts were really present. Omission of non-phytoplankton lineages and manual curation of the taxonomic path ensured optimal phytoplankton characterization based on the 16S region of chloroplasts.

### Data Archiving

In compliance with the Minimal Information about any (X) Sequence (MIxS) standard (Yilmaz et al., 2011), the raw data of demultiplexed and primer-clipped sequences were deposited at the European Nucleotide Archive (ENA; Toribio et al., 2017) using the data brokerage service of the German Federation for Biological Data (GFBio; Diepenbroek et al., 2014). They are accessible under PRJEB26997.

### Statistical Analyses

All statistical analyses were run in R studio using the core distribution (R-Core-Team, 2015) and additional packages, such as *vegan* (Oksanen et al., 2015) and *gplots* (Warnes et al., 2016).

Environmental parameters were displayed in a principal component analysis (PCA). The three missing values of turbidity, fluorescence, and O_2_ were substituted by the means of all other samples of the respective parameter. Based on the PCA, the samples were classified into one of the following categories: surface upwelling (Sur-UW), surface oligotrophic (Sur-oligo), DCM, 200 m, as well as eddy surface (Eddy-sur), eddy DCM (Eddy-DCM), and Eddy-200 m samples. Eddy samples were too different from the others in the respective category but since only one eddy was sampled the Eddy categories had to be excluded from any statistical analyses.

PERmutational Multivariate Analysis Of Variance (PERMANOVA) and Analysis Of SIMilarity (ANOSIM) based on Jaccard dissimilarity were used to test for differences in phytoplankton presence/absence between the categories.

Differences between FL and PA bacterial cell numbers and alpha diversity were assessed with paired *t*-tests. Alpha diversity was assessed using the Inverse Simpson Index calculated without prior rarefying to equal library sizes as rarefaction curves based on this index were saturated at the obtained sequencing depths (Chao et al., 2014). To test for significant differences in alpha diversity among FL and PA, two alternatives were tested: (i) differences occurring between categories of samples identified in the PCA using ANOVA and if applicable Tukey’s test, and (ii) correlation with environmental parameters using a linear model. For all regression-based analyses, collinearity between predictor variables was checked and avoided so that only temperature, salinity, fluorescence, ammonium, and DOC remained.

Pairwise BC dissimilarities were used for cluster analysis (unweighted pair-group method using arithmetic average, UPGMA). Ordination of similarities within each separate fraction was carried out using non-metric multidimensional scaling (NMDS) on relative abundances of FL and PA OTUs. In order to test differences in beta diversity trends between FL and PA, a Procrustes analysis was run on these NMDS ordinations.

Redundancy analysis (RDA) was performed with centered log-ratio-transformed sequence counts of both FL and PA fractions, separately. Variation partitioning was used to assess the contribution of each of the environmental parameters in the RDA models, while accounting for the variability explained by the others (pure effects). Forward model selection was conducted to identify the best-fitting RDA models based on the minimum Akaike Information Criterion (AIC).

## Results

### Physical and Chemical Environmental Parameters

**FIGURE 2:**
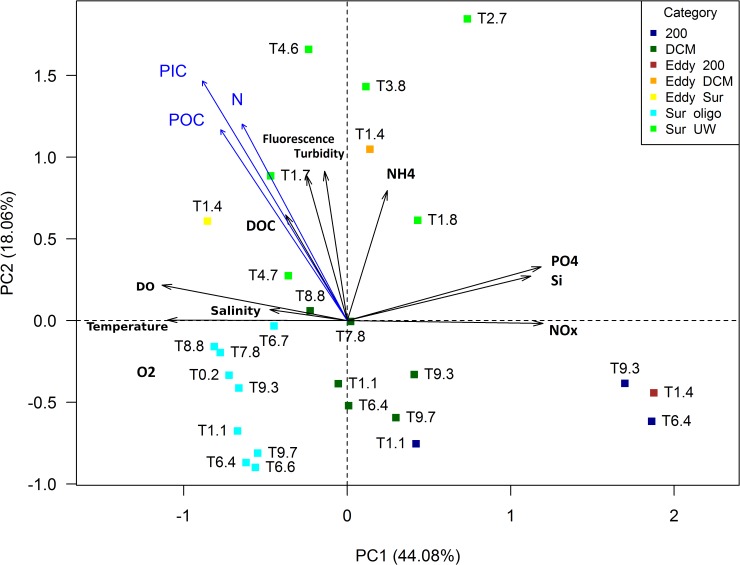
*Principal component analysis (PCA)* of environmental variables measured at the sampling stations (type II scaling). Particulate organic/inorganic carbon (POC/PIC) and particulate nitrogen (N) concentrations were only available from surface waters. They were added to the PCA using *envfit*. DO, dissolved oxygen.

Physicochemical environmental parameters were ordinated in a PCA (Figure 2) and all raw values are archived at PANGAEA^1^ and Supplementary Table 1. In the PCA, the first two principal components explained 62% of the variability in the data set. PC1 was mainly influenced by nutrients (except for ammonium), which were positively correlated with PC1, and physico-chemical parameters related to different water masses, which showed a negative correlation with this PC. PC1 was an indicator for water depth, generally ordinating the deeper stations, which were colder and richer in nutrients, in the positive range. Apart from three upwelling stations (T1.8, T2.7, and T3.8) all surface stations, characterized by higher temperatures and lower nutrients, clustered in the negative range of PC1. PC2 was an indicator for upwelling and was positively correlated with fluorescence and turbidity, as well as DOC and ammonium, i.e., variables indicating productivity.

Three of the four 200 m samples clustered closely together. T1.1 was not grouped closely with the other 200 m samples, indicating its origin from a different water mass. An oxygen minimum with 7 μmol L^-1^ at 30 m depth, the direction of rotation and density structure (obtained from CTD profiles and acoustic doppler current profiler; data unpublished) suggested that station T1.4 was located within an anticyclonic mode water eddy (ACME). The Eddy-DCM station was very different from the remaining loosely clustering DCM stations. At station T1.4, turbidity and fluorescence were highest (0.2 and 6.1 mg m^-3^, respectively) among all DCM samples and were within the range of the Sur-UW samples. Station T1.4 also had the highest N:P ratio (14.5) while all other DCM stations showed ratios between 2 and 12.7.

Six surface stations were characterized by elevated chl*a* concentrations (Figure 1) together with nitrate concentrations of >2 μM and were thus categorized as upwelling samples. Station T2.7 was found on the projectory line of the ammonium vector because it had the highest ammonium concentration of all stations (Figure 2). Temperatures of the Sur-oligo stations were warmer and increased from Mauritania (approximately 23°C) toward Senegal (approximately 29°C). Salinities of Sur-oligo stations ranged from 35 to 37.1 with the lowest and highest values off Senegal (T3.9) and offshore off Mauritania (T0.2), respectively.

Neither of the Sur-UW nor the Sur-oligo surface samples had N:P ratios (all <11) close to Redfield ratio. Nutrient concentrations indicated that the upwelling stations were at most mesotrophic and Sur-oligo stations were oligo- to ultraoligotrophic.

### Phytoplankton Composition

Microscopic analysis (Supplementary Figure 1) of phytoplankton communities at 14 out of 27 samples revealed concentrations between 10,400 cells L^-1^ (at T1.1 200 m) and more than 10^6^ cells L^-1^ (at the Eddy surface station T1.4). The lowest surface numbers were observed at T1.1 with approximately 20,000 cells L^-1^. In general, cyanobacteria and haptophytes were the dominant phytoplankton groups in this area. Diatom cell numbers did not exceed 45,000 cells L^-1^ but were the most diverse phylum (with 20 genera). They included, e.g., *Thalassiosira* and *Chaetoceros*. *Emiliania* and *Phaeocystis* were the only two detected genera of haptophytes.

The presence/absence of phytoplankton genera based on chloroplast sequences (Supplementary Figure 2) indicated spatial trends over the sampling area: Few to no genera were found in the 200 m samples. Upwelling samples clustered together in the heat map, indicating similar phytoplankton communities. Phytoplankton counts at the two Sur-UW stations T3.8 and T4.6 revealed that coccolithophores followed by diatoms and other flagellates were the dominant plankton groups in these samples. Although the classification of samples into the four sample categories was able to significantly explain a small proportion of phytoplankton community variation (PERMANOVA, *F*_(2,21)_ = 1.84, *R*^2^ = 0.15, *p* = 0.032), this effect was not sufficient to result in well-separated communities (ANOSIM, *R* = 0.05, *p* = 0.3).

### Cell Numbers of Bacterial Fractions

**FIGURE 3:**
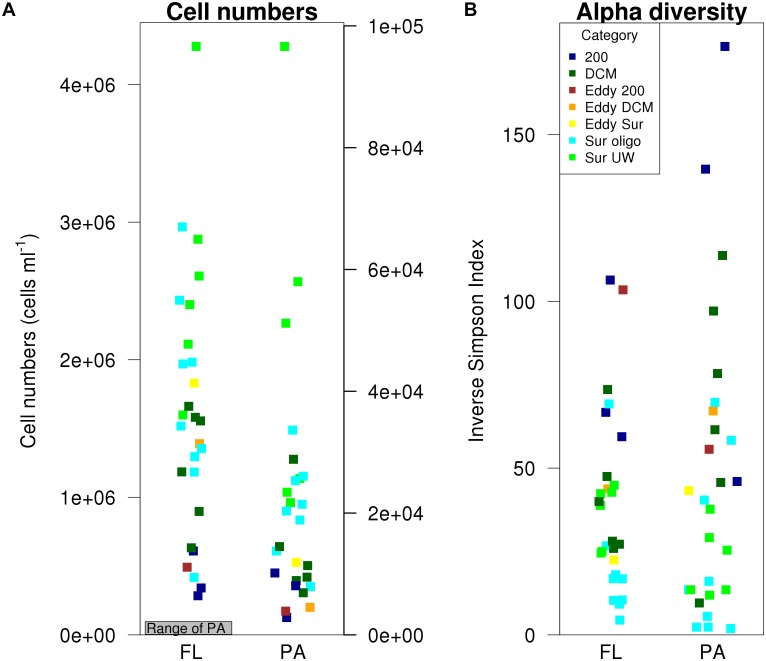
FL and PA cell numbers and inverse Simpson index.

Abundances of FL bacteria were two to three orders of magnitude higher than those of PA (Figure 3). A paired *t*-test confirmed significant differences between FL and PA bacterial abundances (paired *t*-test: *t* = 8.95, *df* = 26, *p* < 0.001). Lowest cell numbers (Figure 3) were generally found in the 200 m samples, although the increase of cell numbers toward the surface stations was more pronounced in the FL fraction. Highest FL cell numbers (>4 × 10^6^ cells mL^-1^) were found at the coastal and nutrient-rich Sur-UW station T3.8. The lowest PA numbers, with <3000 cells mL^-1^, were measured at the deep T1.1 200 m and the saline T0.2 offshore stations, while highest PA bacterial abundances (>9 × 10^5^ cells mL^-1^) were found at the more nutrient-rich and coastal surface stations T4.6 and T4.7.

### Alpha Diversity of Bacterial Fractions

No significant differences in alpha diversity were found between FL and PA (paired *t*-test: *t* = -1.07, *df* = 26, *p* = 0.3). In both fractions, alpha diversity tended to increase with depth. Lowest alpha diversity was found in the Sur-oligo samples. The categories significantly explained underlying patterns in alpha diversity of both FL (ANOVA, *F*_(3,20)_ = 7.79, *p* = 0.001) and PA bacteria (ANOVA, *F*_(3,20)_ = 8.35, *p* < 0.001). Multiple pairwise comparisons indicated that the alpha diversity of FL bacteria at 200 m was significantly different from all other categories (Supplementary Table 3), while in the PA fraction only 200 m and surface communities differed in alpha diversity (Supplementary Table 4). Temperature was identified as the best predictor for explaining differences in alpha diversity in the FL (ANOVA, *F*_(1,22)_ = 22.32, *p* < 0.001) and PA fraction (ANOVA, *F*_(1,22)_ = 8.58, *p* = 0.008).

### Bacterial Community Composition (BCC)

The most common bacterial phyla in this study were Proteobacteria (42%), Cyanobacteria (20%), Bacteroidetes (15%), Actinobacteria (12%), Planctomycetes (5%), Verrucomicrobia (2%), and Marinimicrobia (1.8%).

**FIGURE 4:**
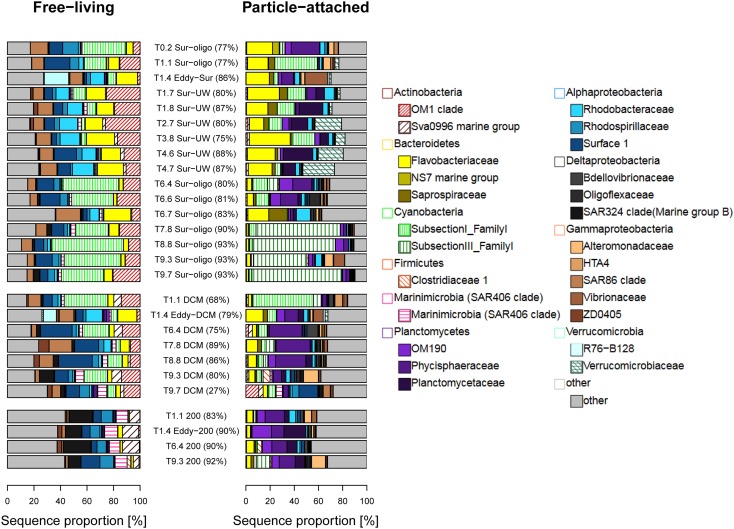
Taxonomic compositions (family level) of FL and PA bacteria. Bray Curtis dissimilarity coefficients are given in brackets.

Actinobacteria, especially the OM1 clade, were typically found among the FL bacteria and shifted from the OM1 group (present in surface and DCM samples) to the family Sva0996 marine group at 200 m (Figure 4). Bacteroidetes, mainly Flavobacteriaceae and Alphaproteobacteria, occurred in both FL and PA fractions. Bacteroidetes dramatically decreased in their relative abundances with depth, especially in the PA fraction off the coast of Senegal. Cyanobacteria had high relative abundances in the surface stations off Senegal. Different types of cyanobacteria dominated the FL and PA communities in the surface stations off Senegal. The FL fraction contained high relative abundances of cyanobacteria belonging to subsection I (e.g., *Synechococcus* and *Prochlorococcus*), while the PA fraction was largely dominated by cyanobacteria belonging to subsection III (mainly *Oscillatoria*). The relative abundances of Cyanobacteria decreased with increasing depth. Marinimicrobia and Planctomycetes tended to increase with depth in the FL and PA fraction, respectively. The family Planctomycetaceae was enriched in the Sur-UW samples, while other families of the same phylum prevailed in the remaining samples (Figure 4). Rhodobacteraceae and Flavobacteriaceae increased in relative abundances in the FL fraction of the upwelling samples. Gammaproteobacteria were represented in both FL and PA fractions, although with different families. Deltaproteobacteria had higher relative abundances at 200 m compared to the surface samples. The highest relative abundances of Verrucomicrobia in this study were found in the Sur-UW samples (from T2.8 to T4.7).

### Beta Diversity and SIMPER

Unweighted pair-group method using arithmetic average (Supplementary Figure 3) clearly separated FL and PA communities with the exception of one PA sample (T9.7-DCM), which clustered with the FL fraction. Pairwise Bray Curtis dissimilarities between 67% and 93% confirmed that FL and PA communities were generally very different. In particular, OTUs of Actinobacteria (OM1 clade) and Cyanobacteria [*Prochlorococcus*, *Synechococcus* (both FL), and *Oscillatoria* (PA fraction)] contributed to the observed differences between FL and PA communities at the surface (Supplementary Figure 4). Off the coast of Senegal (T7–T9) they explained between 40% and 60% of the detected dissimilarity, while off Mauritania they explained less. Dominance of individual OTUs decreased (as alpha diversity increased) with depth. OTUs of Delta- and Gammaproteobacteria were found among the OTUs explaining the detected differences at depth.

Procrustes analysis carried out on separate NMDS ordinations of FL and PA communities (Supplementary Figures 3B,C) suggested that although FL and PA communities might consist of different taxa, their beta diversity patterns were congruent (correlation = 0.8; m12 squared = 0.3529; *p* = 0.001).

### Environmental Drivers Shaping FL and PA Bacterial Communities

The RDA model only using temperature as predictor variable was best suited to explain differences in BCC for the FL fraction (RDA, AIC = 202.79, adjusted *R*^2^ = 0.24, *F*_(1,22)_ = 8.24, *p* = 0.001). For PA bacteria, temperature [adjusted *R*^2^ = 0.082, *F*_(1,21)_ = 3.07, *p* = 0.001] and ammonium [adjusted *R*^2^ = 0.056, *F*_(1,21)_ = 2.41, *p* = 0.001] significantly explained the underlying patterns [RDA, adjusted *R*^2^ = 0.131, *F*_(2,21)_ = 2.74, *p* = 0.001].

**FIGURE 5:**
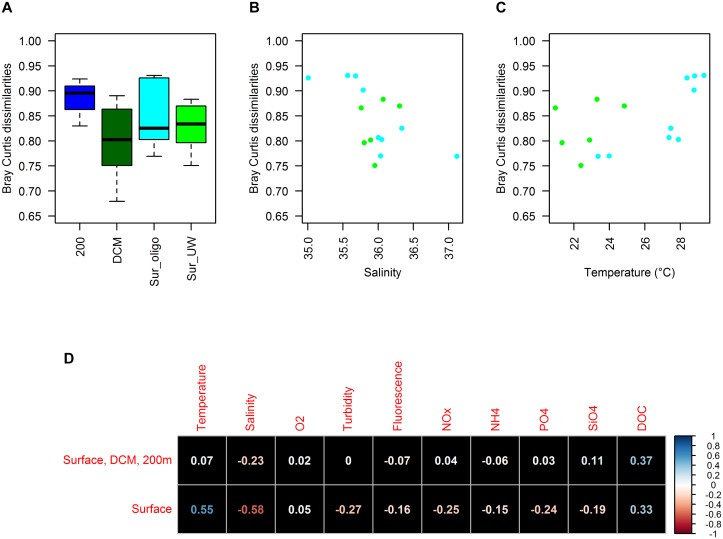
Patterns of pairwise Bray Curtis dissimilarities between FL and PA communities: Differences between sample categories **(A)**, correlation with salinity **(B)** as well as temperature **(C)** for surface samples (especially Sur-oligo), correlation with all observed environmental parameters **(D)**.

Pairwise Bray Curtis dissimilarity coefficients were used to assess changes in the differences between FL and PA communities among categories and environmental parameters. Although insignificant (Kruskal–Wallis, *X*^2^ = 3.45, *df* = 2, *p*-value = 0.18), there was a trend indicating changes in Bray Curtis dissimilarities between FL and PA communities with depth (Figure 5A): While PA and FL communities were most similar at the DCM, Bray Curtis dissimilarities increased to around 90% at 200 m, suggesting major differences between PA and FL communities in deeper water masses. Among surface samples, differences in Bray Curtis dissimilarities existed, even though the surface categories did not explain the underlying changes. While we had insufficient statistical power to test for any significant correlations (Figure 5D), there was an indication that temperature and salinity correlate with surface Bray Curtis dissimilarities (Figures 5B,C). Especially, Bray Curtis dissimilarities of surface oligotrophic samples alone had higher correlations with both salinity (*r* = -0.77) and temperature (*r* = 0.83). Collinearity between temperature and salinity was low (*r* = -0.29) but there was a correlation between NO_x_^-^ and temperature (*r* = -0.73) among the surface samples.

## Discussion

This study provides the first detailed phylogenetic characterization of the BCC off the coast of Mauritania and Senegal. Knowledge about the BCC and especially the PA fraction is the basis for further studying and understanding microbial processes related to the export and recycling of OM in this economically important upwelling system. Our results indicated that differences between FL and PA bacterial communities were simultaneously influenced by environmental parameters and substrate availability.

### Environmental Variables, Phytoplankton, and Bacterial Communities Differ in the Upwelling Region

Surface upwelling and (ultra-)oligotrophic sampling stations have been differentiated using chl*a* and nitrate concentrations. During the boreal summer/autumn, upwelling off NW Africa only occurred North of 21°N as supported by satellite imagery (Figure 1) during our sampling campaign, and water masses most likely had a South Atlantic origin (Schütte et al., 2016). Stations T1.7 and T1.8 were situated at the southernmost tip of the upwelling region. Stations T2.8, T3.8, T4.6, and T4.7 were further south, but due to similar fluorescence values to T1.7 and T1.8, they were also characterized as upwelling samples. Dust input is also an important nutrient source, especially of phosphorous, iron, and other micro- and macronutrients, in this region (Bory and Newton, 2000; Iversen et al., 2010; Lekunberri et al., 2010; Pohl et al., 2011) and may have contributed to elevated nutrient concentrations. The remaining surface samples were characterized by lower fluorescence values. Increasing temperatures (distance to cold upwelling water and proximity to equator) and decreasing salinity (heavy rainfall and proximity to river discharge) occurred from North to South. These Sur-oligo samples have diverse origins, from offshore waters off Mauritania to coastal waters off Senegal.

Phytoplankton communities were dominated by cyanobacteria and coccolithophores, with diatoms peaking at the upwelling stations and T0.2. The dominance of generally tiny phytoplankton cells (coccolithophores and cyanobacteria) may result from low silicate concentrations (Fischer et al., 2009) combined with the oligotrophic conditions, in which smaller cells have an advantage by being able to take up nutrients more efficiently than larger cells (Irwin et al., 2006). The bacterial communities in the ultra-oligotrophic waters off Senegal were characterized by higher relative abundances of Cyanobacteria. There, the ability to fix nitrogen may give cyanobacteria competitive advantages (Carpenter and Price, 1976).

The BCC in the upwelling area off Mauritania was dominated by Alphaproteobacteria, Bacteroidetes, Planctomycetes, and Verrucomicrobia. Planctomycetes, Verrucomicrobia, and some Bacteroidetes (e.g., *Winogradskyella*) are known to be particle-degraders and can therefore be expected to represent key players in the recycling of carbon and other nutrients. The bacterial communities in ultra-oligotrophic waters off Senegal were characterized by higher relative abundances of Cyanobacteria. Additionally, low nutrient conditions and light may have stimulated the growth of FL proteorhodopsin-containing Flavobacteria (Williams et al., 2012). Together, this may explain why Bacteroidetes are present in both fractions (although represented by different genera) and why they tend to occur at higher relative abundances only in surface waters.

### FL and PA Communities Are Influenced by Environmental Parameters

Many studies have demonstrated a significant correlation between BCC and environmental parameters (Hanson et al., 2012 and references therein). In this study, temperature in combination with ammonium or alone was able to explain around 13–24% of PA and FL community variation, respectively. This is in line with previous studies, which suggest that globally temperature is the most important factor for shaping bacterial community structure in epipelagic layers (Sunagawa et al., 2015). The results of our study also indicate that a higher proportion of FL variation can be explained by environmental parameters (in this case temperature) compared to PA bacteria. This can be expected as particles, to which PA bacteria are attached, may act as a “buffer” or micro-island (Lyons et al., 2010; Yung et al., 2016), while FL cells are more directly exposed to the surrounding water. Thereby, the size of the particle is important, because the larger the particle, to which bacteria attach, the more insensitive the bacteria might be to changes in the surrounding water (Yung et al., 2016). Additionally, the high density of bacteria on particles can lead to efficient signaling and quorum sensing (Gram et al., 2002), and nutrient ratios within particles may be very different from those in the surrounding sea water. All these factors may explain why PA bacteria are less affected by changes in environmental parameters than FL bacteria.

### How Can Environmental Parameters Influence the Similarity/Dissimilarity of FL and PA Bacteria?

In this study two environmental parameters, temperature and salinity correlated with changes in Bray Curtis dissimilarities between FL and PA communities; especially when the surface upwelling samples (T1.7–T4.7; Figure 1) influenced by deeper water masses were excluded, and only Sur-oligo samples were taken into account. This correlation may be related to a steady temperature increase toward the equator and lower salinities due to heavy rain fall and riverine water input off Senegal. This in turn may have stimulated the genus *Oscillatoria*, which occurred at high relative abundances in the PA fraction off Senegal. *Oscillatoria* are found at intermediate salinities and are actually filamentous bacteria (Tomitani et al., 2006). While they may be PA, we suggest that they rather represented the particles themselves in this study as we observed filaments in the waters off Senegal, which were visible with the naked eye. This could even suggest that the filamentous bacteria were *Trichodesmium*, which are part of *Oscillatoria*. It has been observed previously that salinity can affect the differences between PA and FL communties, although in contrast to our results, these observations indicated higher similarities as salinity decreased (Ortega-Retuerta et al., 2013). This suggests that salinity may affect the extent to which PA and FL differ. Whether oligohaline vs. marine conditions render the two fractions more similar or dissimilar may, however, also depend on other factors.

### How Can Substrate Availability Affect the Similarity/Dissimilarity of FL and PA Bacteria?

Our results show a trend, indicating that over depth, the Bray Curtis dissimilarities between FL and PA are lowest at the DCM. Although with a cruder method (CARD-FISH), focusing on larger particles, and a different depths resolution, Thiele et al. (2015) confirmed that aggregate-attached communities are most similar to the FL communities at the DCM in the same region but further offshore.

In our study both chl*a* and nutrient concentrations were elevated at the DCM. This suggests a higher substrate availability for PA and FL bacteria when compared to the oligotrophic surface stations (upwelling stations are left out because they are influenced by DCM waters) and 200 m depth stations. PA bacteria have developed different strategies to survive and thrive, and substrate colonization and resource utilization can be very complex. It has been shown that the type of particle substrate and subsequent trophic interactions during its degradation drive the succession of bacteria on particles (Datta et al., 2016). Additionally, a trade-off between colonization and dispersal of two populations of the same species of bacteria has been unraveled (Yawata et al., 2014). This indicates that PA bacteria can have different strategies to exploit the particle resources. While some are adapted to firmly attaching to a particle to thrive in this new microenvironment as best as possible, others remain flexible in order to detach and move to a new hotspot if they receive luring cues. Among others, these two publications have demonstrated the role that the available substrate plays for primary colonization and how important motility can be for some bacteria to access new particles. Under conditions with high substrate availability (as, e.g., prevalent at the DCM) moving between particle hotspots could happen more frequently and could thereby render the PA and FL community more similar. In this context the occurrence of motile PA bacteria in the FL fraction would not imply a specialization for the FL fraction, but would rather stem from their tendency to migrate between hotspots (Yawata et al., 2014).

However, also other reasons are imaginable. For example, high substrate availability at the DCM might be a cue for some PA to detach before they are exported to the deeper oceans, allowing them to stay in the surface layer. Only future studies can elucidate these hypotheses further, but as the life-styles of bacteria coincide with functional differences and therefore implications for biogeochemical cycling, it is worth investigating this topic in more detail.

## Conclusion

This study provides the first detailed description of the BCC off the coasts of Mauritania and Senegal. As this region is not only important for global fisheries but also responsible for a large draw down of carbon, our study is vital for a better understanding of the ecosystem function in this area. The high relative abundances of several groups of Planctomycetes, Verrucomicrobia, and some Bacteroidetes (e.g., *Winogradskyella*) indicated that these microbes may represent key players in the recycling of carbon in this area. Our results can serve as a basis for future research aimed directly at microbial processes related to the export and recycling of OM in the Mauritania upwelling area.

We have demonstrated the importance of systematically distinguishing between FL and PA communities, as the two fractions can be very different in abundance and functionality. Differences between FL and PA bacteria in oligotrophic surface samples correlate with changes in salinity and temperature and the prevalent conditions off Senegal promoted the growth of *Oscillatoria*. Similarities between FL and PA bacteria were highest at the DCM and may suggest that high substrate availability reduces the dissimilarity of the two fractions. This calls for further studies on the influence of environmental parameters and substrate availability on the similarity and dissimilarity between FL and PA fractions of heterotrophic bacteria and their respective biogeochemical functions.

Our study provides a valuable basis for future studies, which should focus on seasonal changes in microbial community structure and related biogeochemical function as well as changes in the similarity/dissimilarity of FL and PA as the upwelling region expands and contracts within the Mauritania upwelling area.

## Data Availability

The environmental dataset generated for this study can be found at PANGEA (https://doi.pangaea.de/10.1594/PANGAEA.889977) and sequencing data are stored at the ENA (accession number: PRJEB26997).

## Author Contributions

JB, HG, MI, and AG conceived and designed the study. JB, TH, and GK performed the field and lab work. JB, TH, and CH performed the data analyses. JB wrote the manuscript. HG, CH, MI, GK, and AG contributed to the final manuscript.

## Conflict of Interest Statement

The authors declare that the research was conducted in the absence of any commercial or financial relationships that could be construed as a potential conflict of interest.

## Footnotes

1 https://doi.pangaea.de/10.1594/PANGAEA.889977
