# Supplementary material for: Environmental Drivers of Free-Living vs. Particle-Attached Bacterial Community Composition in the Mauritania Upwelling System
Source: Front Microbiol. 2018 Nov 23;9:2836. doi: 10.3389/fmicb.2018.02836 (PMC6265507; doi:10.3389/fmicb.2018.02836)
Supplement: Supplementary file 1 [file Data_Sheet_1.docx]

Supplementary Material

Environmental drivers of free-living vs. particle-attached bacterial community composition in the Mauritania upwelling system

Jennifer Bachmann*^1,2^,Tabea Heimbach^1,2,3^, Christiane Hassenrück^1^, Germán A. Kopprio^1^, Morten Hvitfeldt Iversen^4,5^, Hans Peter Grossart^6,7^, Astrid Gärdes^1^

*** Correspondence:** Jennifer Bachmann: Jennifer.bachmann@leibniz-zmt.de

## Supplementary Figures

##
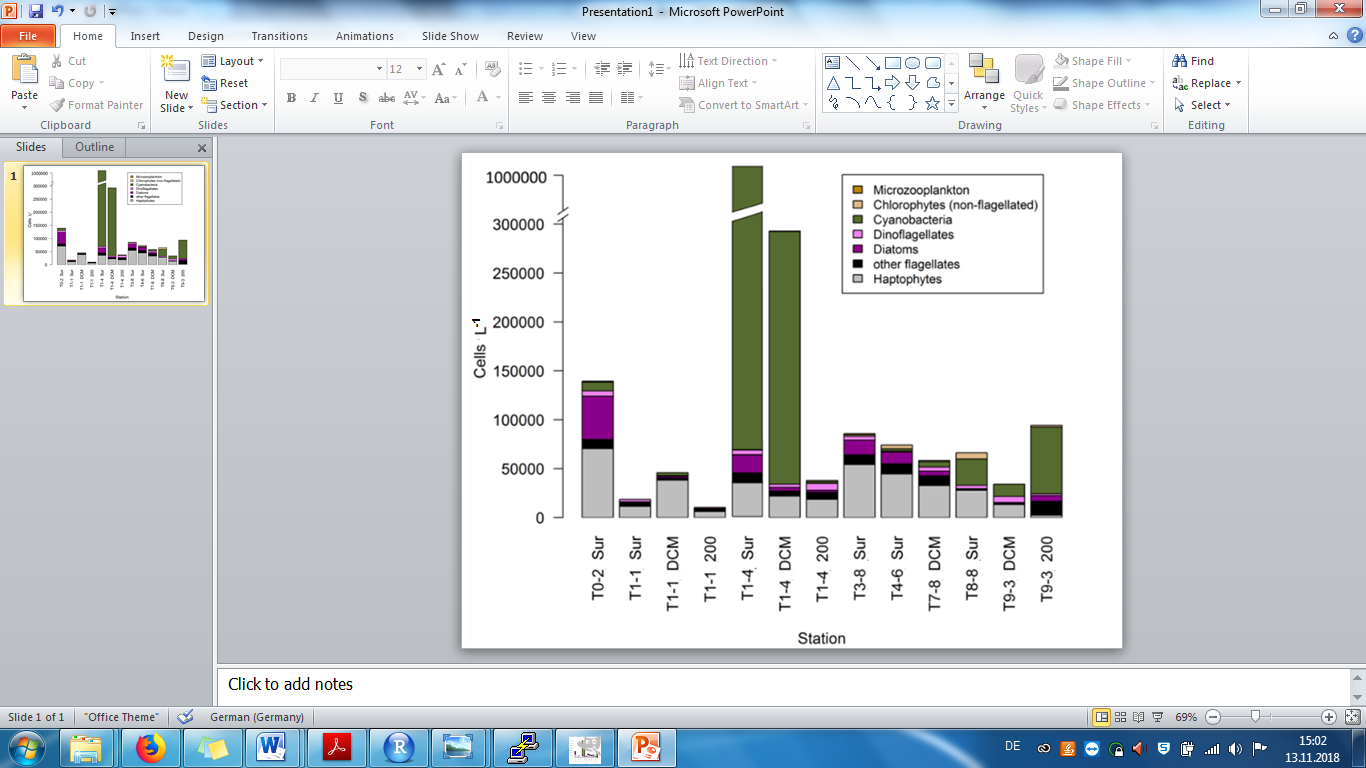


## Supplementary Figure 1. Cell counts of major plankton groups at selected stations.


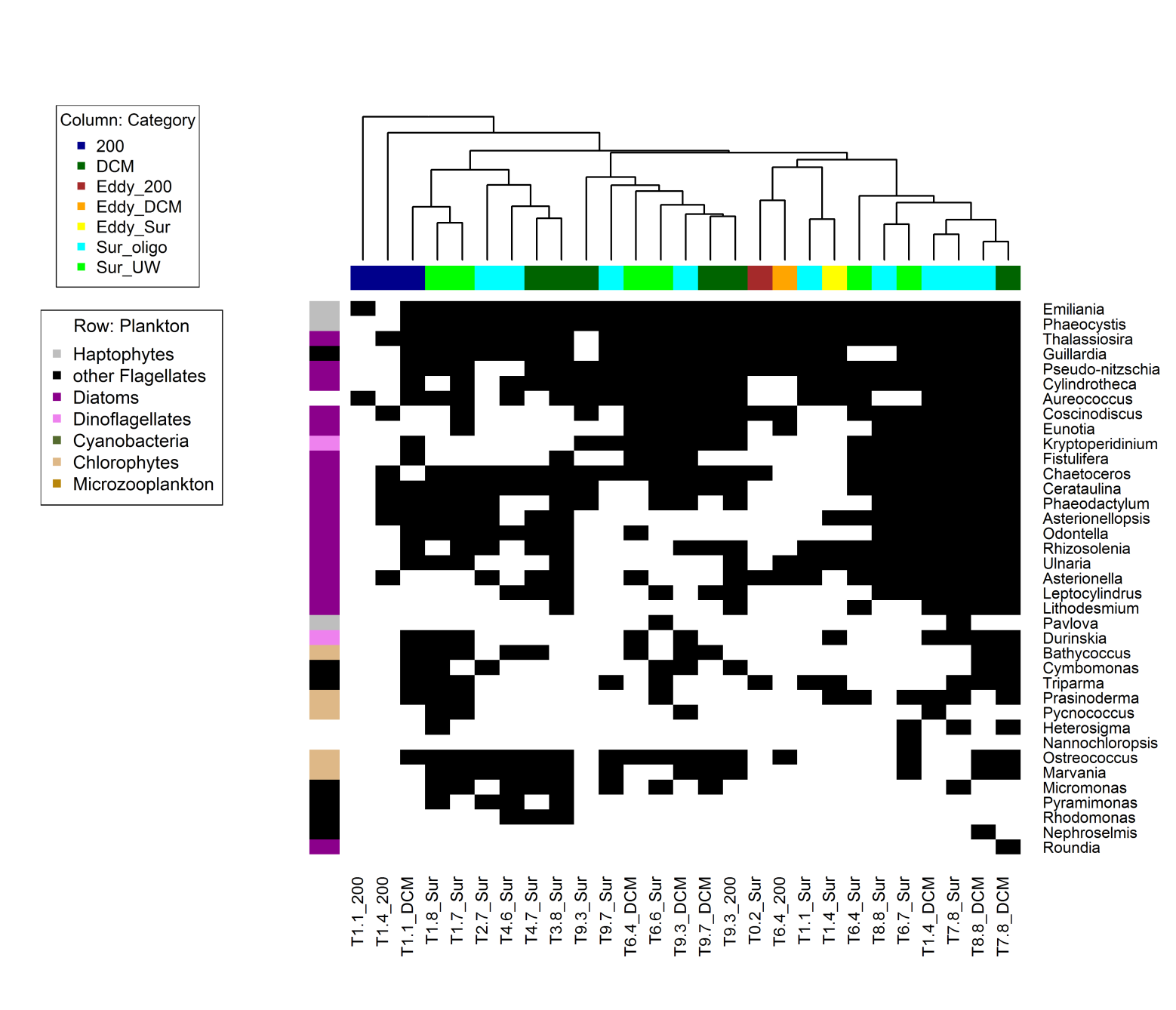
**Supplementary Figure 2.** Presence (black) and absence (white) heat map with all phytoplankton genera. Color code at the top represents the different categories and color code at the left side represents the phytoplankton groups as shown in Supplementary Figure 1.


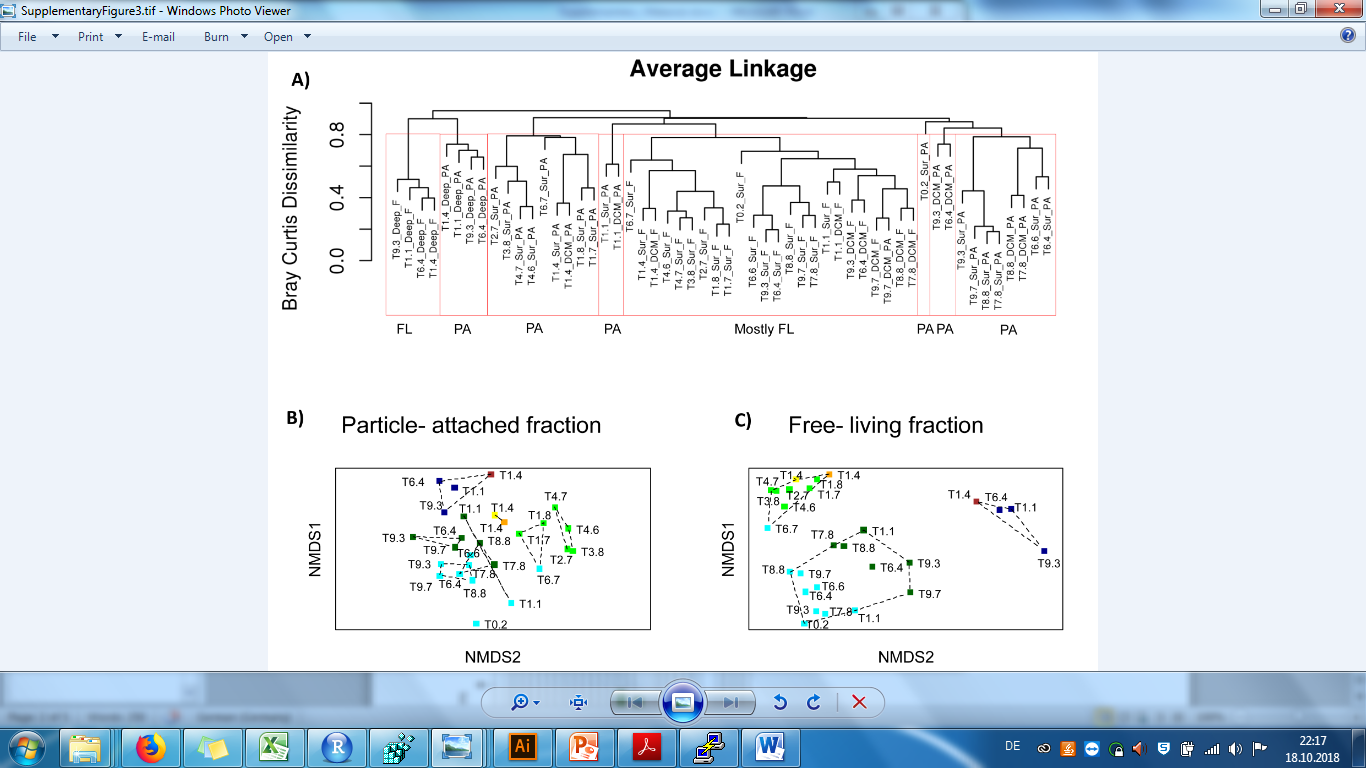


**Supplementary Figure 3.** Unweighted-pair group method with arithmetic mean (A; UPGMA) cluster dendrograms based on Bray Curtis dissimilarities of PA and FL bacteria and non- metric dimensional scaling (NMDS) plots for PA and FL communities (B, C), on which the Procrustes analysis is based. Red squares and hulls indicate 80% similarity.


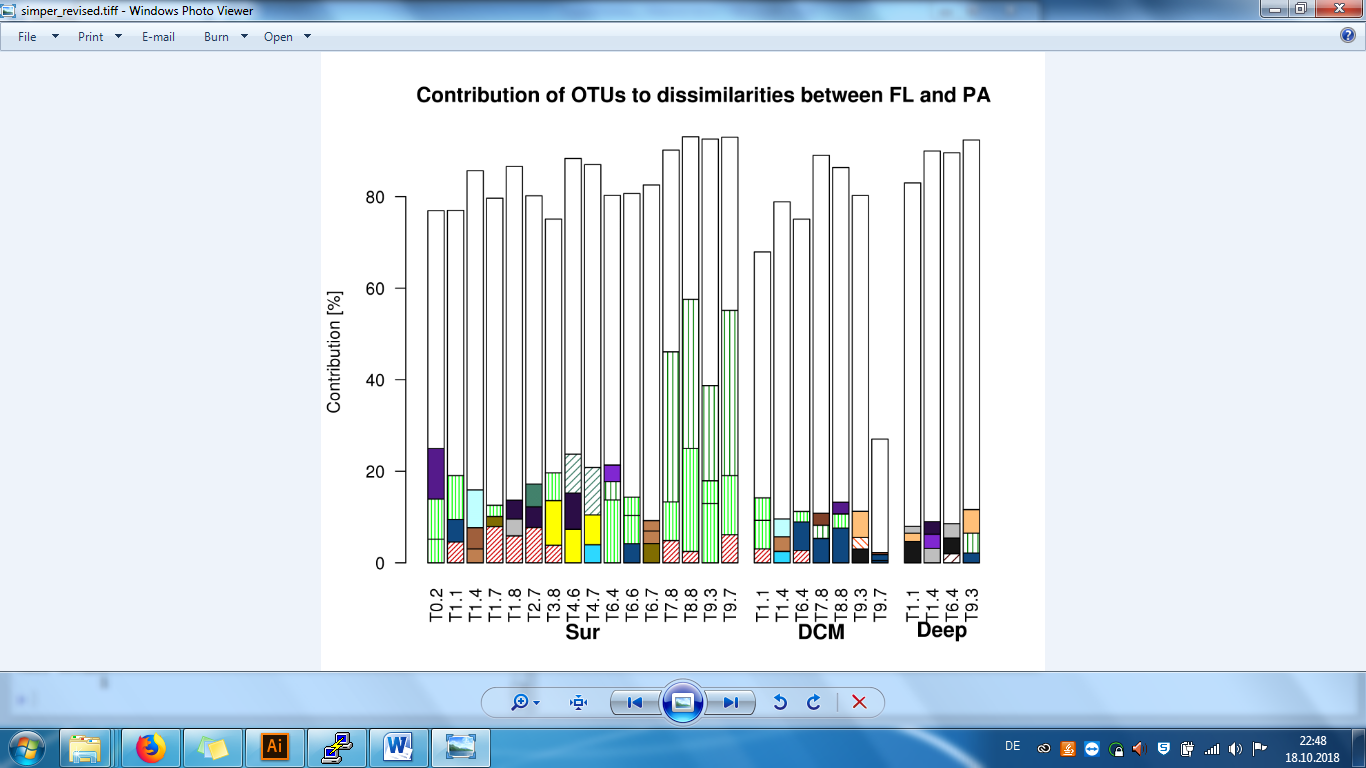


Supplementary Figure 4. Dissimilarity percentages (SIMPER): Contribution of the three most common OTUs to the dissimilarity (Bray Curtis, Figure 4) between FL and PA. For color code see Figure 4.

## Supplementary Tables

**Supplementary Table 1.** Meteor station, station depth, physico-chemical parameters and phytoplankton sampling points (phyto) are displayed.

## Supplementary Table 2. Filtered volume for bacterial community analysis

|  |  | Filtered volume (L) | |
| --- | --- | --- | --- |
| Station | Depth | FL | PA |
| T0.2 | Surface | 1 | 20 |
| T1.1 | Surface | 0.8 | 4 |
|  | DCM | 1 | 4 |
|  | 200 m | 1 | 4 |
| T1.4 | Surface | 0.25 | 1 |
|  | DCM | 0.25 | 1 |
|  | 200 m | 0.75 | 3 |
| T1.7 | Surface | 0.25 | 1 |
| T1.8 | Surface | 0.25 | 1 |
| T2.7 | Surface | 0.25 | 1 |
| T3.8 | Surface | 0.25 | 1 |
| T4.6 | Surface | 0.25 | 1 |
| T4.7 | Surface | 0.25 | 1 |
| T5.5 | Surface | 0.5 | 1.5 |
| T6.4 | Surface | 0.5 | 3 |
|  | DCM | 0.5 | 2 |
|  | 200 m | 0.5 | 2 |
| T6.6 | Surface | 0.5 | 2 |
| T6.7 | Surface | 0.25 | 1 |
| T7-8 | Surface | 0.25 | 2 |
|  | DCM | 0.25 | 1.5 |
| T8-8 | Surface | 0.5 | 2 |
|  | DCM | 0.25 | 1 |
| T9-3 | Surface | 0.25 | 1 |
|  | DCM | 0.5 | 3 |
|  | 200 m | 0.5 | 4 |
| T9-7 | Surface | 0.25 | 2 |
|  | DCM | 0.25 | 2 |

|  |  |  |  |
| --- | --- | --- | --- |

**Supplementary Table 3.** Tukey’s HSD results of the effect of categories on the alpha diversity of FL communities

**p (adj)**

**DCM-200**  0.038

**Sur-oligo-200** 0.0006

**Sur-UW-200** 0.019

**Sur-oligo-DCM** NS

**Sur-UW-DCM** NS

**Sur-UW-Sur-oligo** NS

Supplementary Table 4. Tukey’s HSD results of the effect of categories on the alpha diversity of PA communities

**p (adj)**

**DCM-200** NS

**Sur-oligo-200** 0.001

**Sur-UW-200** 0.002

**Sur-oligo-DCM** NS

**Sur-UW-DCM** NS

**Sur-UW-Sur-oligo** NS
